# Surgically resected T1‐ and T2‐stage esophageal squamous cell carcinoma: T and N staging performance of EUS and PET/CT

**DOI:** 10.1002/cam4.1617

**Published:** 2018-06-22

**Authors:** Dong Young Jeong, Min Yeong Kim, Kyung Soo Lee, Joon Young Choi, Soo Jeong Kim, Myung Jin Chung, Yang Won Min, Hong Kwan Kim, Jae Ill Zo, Young Mog Shim, Jong‐Mu Sun

**Affiliations:** ^1^ Department of Radiology Samsung Medical Center Sungkyunkwan University School of Medicine Seoul Korea; ^2^ Department of Nuclear Medicine Samsung Medical Center Sungkyunkwan University School of Medicine Seoul Korea; ^3^ Division of Gastroenterology Department of Medicine Samsung Medical Center Sungkyunkwan University School of Medicine Seoul Korea; ^4^ Department of Thoracic Surgery Samsung Medical Center Sungkyunkwan University School of Medicine (SKKU‐SOM) Seoul Korea; ^5^ Division of Hemato‐oncology Department of Medicine Samsung Medical Center Sungkyunkwan University School of Medicine Seoul Korea

**Keywords:** early stage cancer, endoscopic submucosal dissection, endoscopic ultrasonography, esophageal cancer, esophageal cancer staging, PET/CT

## Abstract

This study aimed to evaluate the frequency of nodal metastases and to disclose the diagnostic performance of endoscopic ultrasonography (EUS) and PET/CT in T and N staging in surgically resected early‐stage esophageal squamous cell carcinomas (eSCCs). Institutional review board approved this retrospective study with waiver of informed consent for reviewing medical record. We included 435 patients with an early T‐stage (Tis or T1a [≤T1a], T1b and T2) eSCC. The rates of metastatic lymphadenopathy were calculated. Then, the performance of EUS and PET/CT in subdividing T and N stages was assessed. 131 ≤ T1a, 234 T1b, and 70 T2 eSCCs were identified. In discriminating ≤T1a from other cancers, the sensitivity, specificity, and accuracy of EUS were 60.3% (79/131), 80.3% (244/304), and 74.3% (323/435) respectively. With ROC curve analysis, cut‐off value of SUVmax 3.05 at PET provided sensitivity 74.8% (98/131), specificity 70.1% (213/304), and accuracy 71.5% (311/435) for differentiating ≤T1a eSCCs from others. Ten (7.6%) of 131 ≤ T1a cancers had nodal metastasis. In discriminating N0 from node‐positive disease, sensitivity, specificity, and accuracy of EUS were 89.6% (267/298), 41.6% (57/137), and 74.5% (324/435), respectively, whereas those of PET/CT were 88.9% (265/298), 38.7% (53/137), and 73.1% (318/435) respectively. In >70% of patients with ≤T1a eSCCs, the tumor stage can be discriminated from higher stage cancers by using EUS or PET/CT. Substantial percentage (7.6%) of ≤T1a eSCC patients have nodal metastases, which are missed in more than half of the patients in clinical staging.

## 1 INTRODUCTION

Esophageal cancer (ECA) is one of the 10 most prevalent cancers, accounting for 4% of incidence rates among all cancers. The ECA is also one of the top 10 causes of cancer‐related deaths. The most common histopathologic subtype of ECA is squamous cell carcinoma (eSCC; 85% of all esophageal cancers).^1^

Surgery is the treatment of choice in localized early eSCC (T1b‐T2 N0‐1 and M0) and endoscopic submucosal dissection (ESD) is a treatment option for selected patients (TIS [high‐grade dysplasia] or T1a, collectively called ≤T1a eSCC).^2^ Thus, to differentiate T1a eSCC from T1b or higher‐stage eSCCs would help decide management method for T1 eSCCs. On the other hand, in locally advanced eSCCs, preoperative chemotherapy or chemoradiation therapy may help increase the rates of complete resection, thus decreasing local tumor recurrence and improving survival.

Lymph node metastases are more widely distributed in eSCCs, whereas those in esophageal adenocarcinomas (ADCs) are limited to loco‐regional nodes. The rates of lymph node metastases in eSCC are relatively high even in early‐stage eSCCs than those in ADCs.^3^

According to a meta‐analysis study, overall endoscopic ultrasonography (EUS) has good accuracy with pooled sensitivity and specificity for T1a staging of 0.85 (95% confidence interval, 0.82‐0.88) and 0.87 (95% confidence interval, 0.84‐0.90). Heterogeneous results from various studies may derive from multiple factors including the location and type of lesion, method and frequency of EUS probe, and the experience of the endo‐sonographer.^4^ In N staging, regardless of T stages, EUS has low sensitivity (usually less than 50% sensitivity), for detecting metastatic nodes.^5^

To the best of our knowledge, the role of ^18^F‐fluorodeoxy glucose (FDG) PET/CT in differentiating T1a from higher stage eSCCs has not been published. Furthermore, PET/CT was falsely negative in early stage esophageal cancer where nodal metastasis is occasional.^5^, ^6^ Thus, little datum is available on the role of FDG PET/CT in T and N staging in early‐stage esophageal cancers, particularly in eSCCs. Thus, the purpose of this study was to demonstrate the frequency of nodal metastases and to disclose the diagnostic performance of EUS and PET/CT in T and N staging in surgically resected early stage (T1‐ and T2‐stage) eSCCs.

## 2 MATERIALS AND METHODS

Institutional review board (IRB) approved this retrospective study; informed consent for reviewing patients’ electronic medical record was not obtained from each patient.

### 2.1 Patients

**Figure 1:**
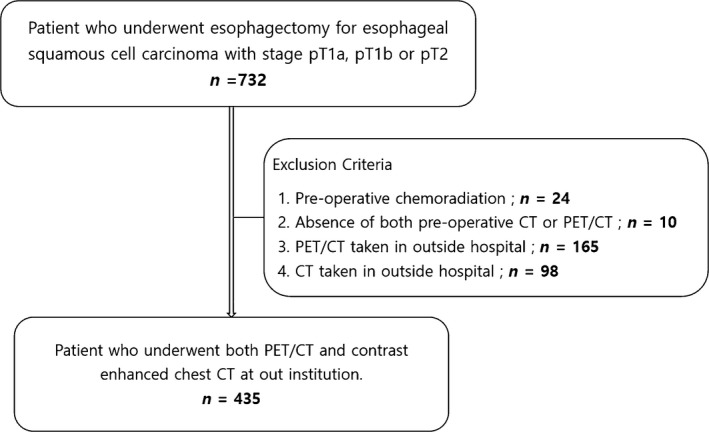
Flowchart showing how patients were included

From January 2010 to December 2016 when surgery and its staging was recorded in consideration of 7th version of esophageal staging system, 1498 patients received esophagectomy and lymph node dissection. Of them, 732 patients proved to have eSCCs of ≤T1a, T1b, or T2 stage. Among them, 297 patients were excluded with the following reasons; 24 patients were excluded owing to neoadjuvant chemoradiation therapy (n* *=* *7) or concurrent chemoradiation therapy (n* *=* *17), 10 patients did not undergo either enhanced chest CT or PET/CT, 165 patients underwent PET/CT in outside hospital (difficulty in measuring maximum standardized uptake value [SUVmax]), and 98 patients underwent chest CT in outside hospital (with incomplete or different CT parameters for the evaluation of esophageal cancer and its staging). Thus, the remaining 435 patients who underwent both PET/CT and chest CT at our institution were finally included in this study (Figure 1).

### 2.2 Endoscopic ultrasonography (EUS)

Endoscopic ultrasonography was performed by one of two physicians (with endoscopic experience of 10 and nine years, respectively) using water‐filling method and adopting a radial scanning catheter probe (12/20‐MHz, UM‐2R/3R, Olympus, Tokyo, Japan). The probe was passed through one instrument channel of a 2‐channel endoscope (GIF‐2T240/GIF‐2TQ260M, Olympus). The second channel was used for the instillation of 100‐200 mL water by using a pump system. The depth of tumor invasion and the lymph‐node status was assessed by cross‐sectional images of the esophageal wall and peri‐esophageal area. Lymph nodes were considered malignant, when it was larger than 10 mm in any axis, hypoechoic, and with rounded shape and clearly defined margin.

### 2.3 CT and PET/CT scanning

CT scanning was performed with various helical CT scanners of four vendor companies (Data S1). Helical CT scans were obtained from the 3 cm above sternal notch to the renal hila with 2.5‐3‐mm detector collimation and a pitch of 0.984‐1.5. The scans were obtained after intravenous injection of a total of 100 mL of contrast medium (Iomeprol [Iomeron 300]; Bracco, Milan, Italy or Iobitridol [Xenetix 300]; Guerbet, Aulnaysous‐Bois, France) at a rate of 1 mL/sec for the first 40 mL and 2 mL/s for the remaining 60 mL with a power injector (MCT Plus; Medrad, Pittsburgh, Pa). Esophageal distension with the administration of water and effervescent granules or air insufflation was not performed. Scanning parameters were 120 kVp and 114‐275 mA under automatic exposure control; beam width, 10‐20 mm; rotation time was 0.3‐0.4 seconds. Image data were reconstructed by both transverse and coronal planes with a soft tissue algorithm and with 2.5‐mm‐thickness and at the same‐thickness intervals. The reconstructed image data were directly interfaced and sent to picture archiving and communication system (Centricity 3.0; GE Healthcare, Mount Prospect, IL, USA) which displayed all image data on two monitors (1536 × 2048 matrix, 8‐bit viewable gray scale, and 60‐ft‐lambert luminescence). Both mediastinal (width, 400 HU; level, 20 HU) and lung (width, 1500 HU; level, −700 HU) window images were viewed on these monitors.

All patients fasted for at least 6 hours before PET examination. Blood glucose levels were measured before injection of FDG and were required to be <150 mg/dL in all patients. Whole‐body PET and unenhanced CT images were acquired using two kinds of PET/CT scanners (Discovery LS, GE Healthcare, Milwaukee, WI, USA; Discovery STe, GE Healthcare, Milwaukee, WI, USA), 60 minutes after the injection of FDG (5.5 MBq/kg). When the Discovery LS scanner was used, whole‐body CT was performed with a continuous spiral technique with an 8‐slice helical CT (140 keV; 40‐120 mA; section width, 5 mm). After the CT scan, an emission scan was obtained from head to middle thigh for 4 min per frame in 2‐dimensional mode. Attenuation‐corrected PET images (4.3 × 4.3 × 3.9 mm) were reconstructed from the CT data using an ordered‐subset expectation maximization (OSEM) algorithm (28 subsets, 2 iterations). When the Discovery STe scanner was used, whole‐body CT was performed with a continuous spiral technique with 16‐slice helical CT (140 keV; 30‐170 mA; section width, 3.75 mm). After the CT scan, an emission scan was obtained from the head to middle thigh for 2.5 minutes per frame in 3‐dimensional mode. Attenuation‐corrected PET images (3.9 × 3.9 × 3.3 mm) were reconstructed from the CT data using a 3‐dimensional OSEM algorithm (20 subsets, 2 iterations). Standardized uptake value (SUV) was derived from the injected dose of FDG and the patient's body weight.

### 2.4 CT and PET/CT study interpretation for T and N staging

One of two radiologists (with two‐year‐experience of thoracic CT interpretation and with 4‐year‐experience of both thoracic and abdominal CT interpretation, when they first read the CT studies) who were blinded to the results of PET/CT and pathologic results but aware of having an esophageal cancer, prospectively interpreted the CT images. Each radiologist analyzed CT images and recorded the presence or absence of abnormalities in the esophagus; no identifiable tumor along the entire course of the esophagus (≤T1a stage), or definitely visible wall thickening or tumor with >5 mm in thickness or tumor diameter but less than 10 mm (T1b stage), with 10‐15 mm in thickness or tumor diameter (T2), and with >15 mm in thickness or tumor diameter (T3). When identifiable esophageal lesions were present, the location and length of the lesions were recorded in consideration of four anatomic landmarks: thoracic inlet, azygos arch, inferior pulmonary veins, and the esophago‐gastric junction.

Lymph nodes were classified into the following 11‐group nodal stations according to a modified version of the lymph node mapping system for esophageal cancer proposed by Korst et al^7^ group Cx, cervical; group Pt, paratracheal; group 5, aortopulmonary; group 7, subcarinal; group 8, paraesophageal; group 9, inferior pulmonary ligament; group 10, hilar; group 15, diaphragmatic; group 17, left gastric; group 18, common hepatic; and group 20, celiac. In addition, lymph nodes at gastric cardia were classified into para‐cardial group. Intrathoracic and abdominal lymph nodes >10 mm in short‐axis diameter, supraclavicular nodes >5 mm in diameter, and retrocrural nodes more than 6 mm in diameter were regarded as abnormal lymph nodes.^2^

One of two nuclear medicine physicians (16 years and 10 years of experience in PET/CT interpretation, respectively) and one chest radiologist (26 years of chest CT interpretation and 10 years experience of PET/CT interpretation), who were blinded to clinical and pathologic results, prospectively evaluated PET/CT in consideration of chest CT scan results. As for tumors, SUVmax was measured at tumor sites. When the primary cancer was not visualized or could not be distinguished from the background (n* *=* *70), SUVmax was assigned an assumed default value of 1.0 similar to background uptake. Regarding nodal staging, the area of FDG uptake at PET greater than mediastinal blood pool uptake where identifiable lymph nodes were present at CT, was considered to be positive for malignancy. Any lymph nodes, when they were higher in attenuation (>70 HU) than mediastinal structures or containing calcifications on unenhanced scans, were considered negative for metastasis at PET/CT, even though they showed higher FDG uptake at PET than mediastinal blood pool or SUVmax greater than 3.5.^8^

### 2.5 Surgery and pathologic specimen analyses

Patients with the primary tumor involving the cervical or upper thoracic esophagus (azygos arch or above, n* *=* *49) underwent transthoracic esophagectomy (involving laparotomy, right thoracotomy, and cervical anastomosis) with LN dissection in three fields (thoracic, abdominal, and cervical [including supraclavicular nodes]). Patients with the primary tumor located in the middle portion of the esophagus (below the azygos arch down to the right inferior pulmonary vein, n* *=* *169), the lower thoracic esophagus or gastroesophageal junction (n* *=* *189), and middle to lower thoracic esophagus (n* *=* *28) underwent transthoracic esophagectomy (involving laparotomy, right thoracotomy, and high thoracic anastomosis) with two‐field (thoracic and abdominal) lymph node dissection (Table 1). During surgery, one of three thoracic surgeons (with 31, 28, and 17 years of esophageal cancer surgery experience, respectively), dissected all visible and palpable LNs in the surgical field, taking into consideration all results from the preoperative imaging examinations, including EUS, enhanced CT and FDG PET/CT.

**Table 1:** Demographics, EUS, CT, and pathologic T staging characteristics

Characteristics	pTis + pT1a	pT1b	pT2	Total	*P* value
Gender					
Male	119	218	66	403	.608^a^
Female	12	16	4	32	
Mean age (y)^b^ (min to max)	64 (31‐90)	64 (40‐85)	66 (44‐78)	64 (31‐90)	.400^a^
Locations					
Cervical	0	1	0	1	.147^a^
Upper thoracic	14	16	5	35	
Mid thoracic	50	99	20	169	
Lower thoracic	52	102	35	189	
Upper to mid thoracic	3	6	0	9	
Mid to lower thoracic	10	8	10	28	
Upper to lower thoracic	1	1	0	2	
Upper and lower thoracic	1	1	0	2	
Pathologic T staging					
Tis	5			5	
T1a	126			126	
T1b		234		234	
T2			70	70	
EUS T staging					
Tis	8	9	1	18	<.001^a^
T1a	71	67	2	140	
T1b	41	105	15	161	
T2	11	47	34	92	
T3	0	6	18	24	
CT T staging					
T1a or less	108	167	21	296	<.001^a^
T1b	19	51	11	81	
T2	4	16	37	57	
T3	0	0	1	1	
SUVmax (Q1‐Q3)	2.53 (1.00‐3.10)	4.02 (2.50‐4.80)	9.69 (5.78‐13.4)	4.48 (2.40‐5.40)	<.001^b^
Total	131	234	70	435	

a Calculated with Pearson's chi square test.

b Calculated with ANOVA.

The esophagectomy specimen was opened longitudinally in the fresh state. After periesophageal fat was dissected, LNs were sought. Thereafter, the specimen was fixed overnight in 10% neutral buffered formalin. Descriptions of the tumor (*i.e.,* appearance, depth of invasion, length from both lines of resection and from cardia), the mucosal appearance, the wall thickness, and the lymph nodes (the location, size, and number of nodes seen) were recorded. Sections were obtained for the histopathologic evaluation of the tumor, the non‐neoplastic mucosa, the proximal and distal lines of resection, and the lymph nodes. The specimens were stained with a standard hematoxylin‐eosin technique and examined with light microscopy.

In each patient, the pathologic stage of primary esophageal cancer was recorded. A total 15 432 lymph nodes were dissected and classified as nodal stations.

### 2.6 Statistical analysis

Endoscopic intervention (endoscopic submucosal dissection, ESD) can be indicated for eSCCs confined to the mucosa or lamina propria (T1a or less in stage) without submucosal invasion.^9^, ^10^ Thus, it was very important to know depth of tumor invasion and the presence of lymph node metastasis. In T staging, the distinction capability (sensitivity, specificity and accuracy) of EUS between ≤T1a and T1b or T2 was compared with that of CT or PET.

For the SUVmax of primary tumor, ANOVA test was carried out among three groups (≤T1a, T1b, and T2). Then, the Pearson product‐moment correlation coefficient was calculated for analyzing relationship between SUVmax and pathologic T stages and the receiver operating characteristic (ROC) curves were constructed and depicted in order to obtain the most appropriate cut‐off values in terms of differentiating ≤T1a from T1b or T2 cancers and differentiating ≤T1 and T2.

The sensitivity, specificity, accuracy, positive predictive value (PPV), and negative predictive value (NPV) of EUS and PET/CT in differentiating N0 disease from N1, N2 or N3 disease were calculated on a per‐person basis.

Intermodality agreements on both T and N staging of each modality were calculated by weighted kappa for comparing its performance, because the data range of both T and N staging was not dichotomous.^11^ Both bootstrap method and GEE (generalized estimating equations) were used for calculating confidence interval and comparing correlated kappa coefficients.

All statistical analyses were performed with SPSS software (SPSS for Windows, version 22.0; SPSS, Chicago, IL) and the statistical computing language R (version 3.4.3, R Foundation). Statistically significant differences were defined as having *P* values <.05.

## 3 RESULTS

### 3.1 Demographics and pathologic characteristics

**Figure 2:**
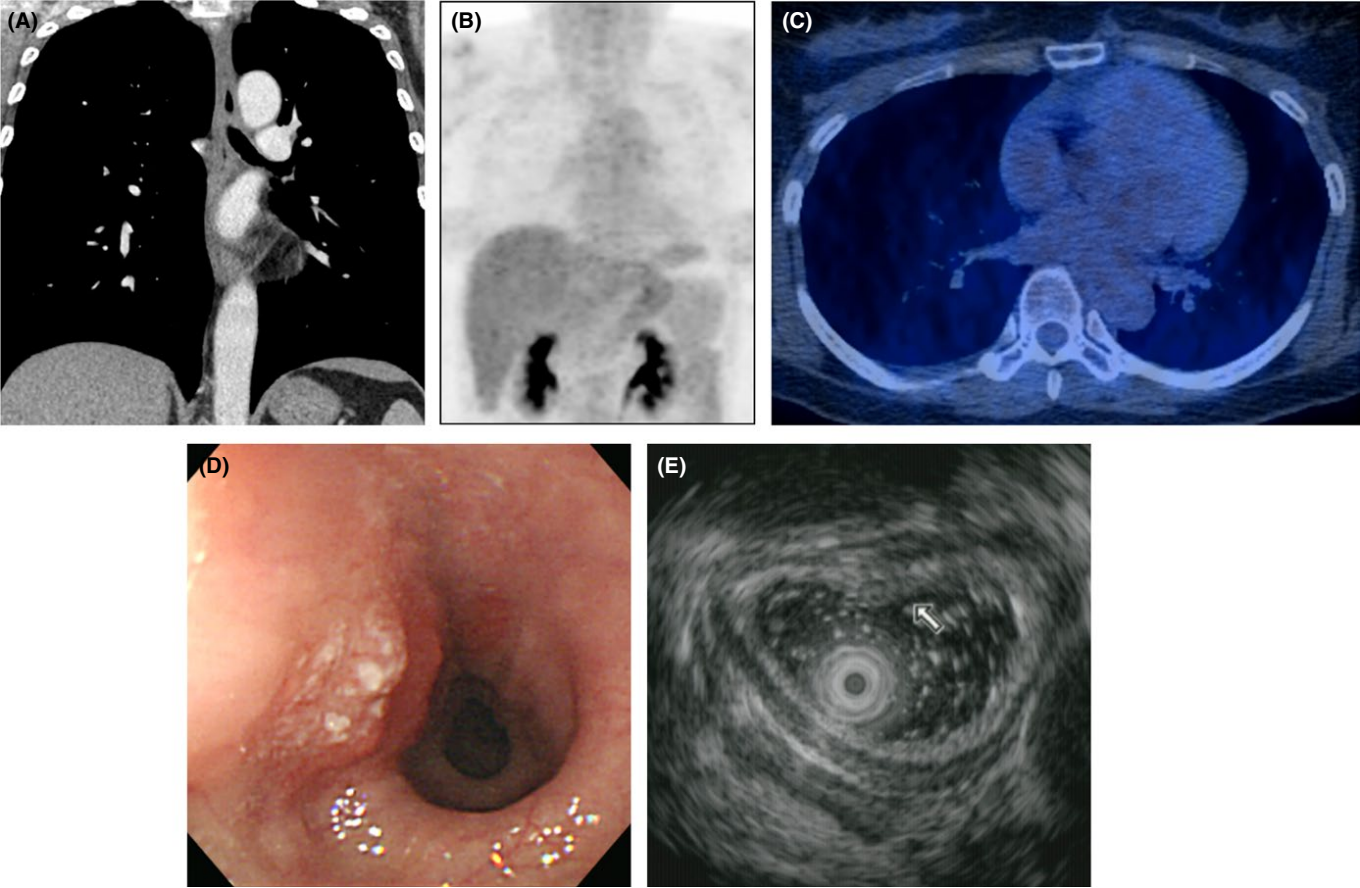
pT1aN0‐stage esophageal squamous cell carcinoma in a 51‐year‐old woman involving the intrathoracic lower thoracic esophagus without lymph node metastasis. (A) Coronal reformatted CT image shows no abnormal wall thickening or mass along the entire esophagus of the esophagus. (B, C) Maximum intensity projection (B) and fused (C) PET/CT image demonstrates no remarkable FDG uptake along the entire course of the esophagus (SUVmax = 2.4 at presumed tumor site). (D) Endoscopy demonstrates a 14‐mm‐sized flat elevated lesion with granular protuberance in the lower esophagus (31 cm from incisor teeth). (E) The lesion is mucosal thickening with preserved submucosal layer (arrow) on EUS. Nodal metastasis was not identified even on pathologic examination

**Figure 3:**
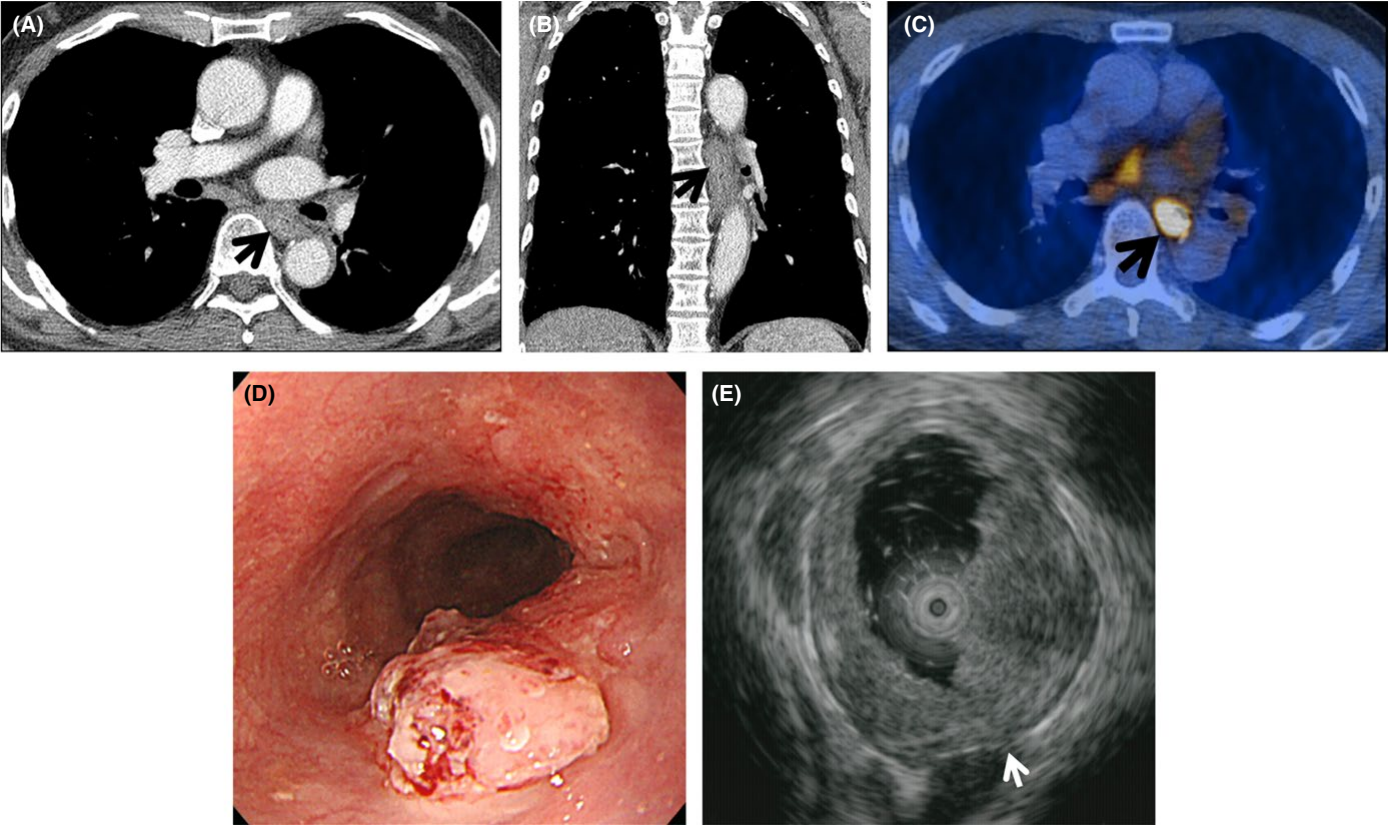
pT2N1‐stage esophageal squamous cell carcinoma in a 74‐year‐old man involving the intrathoracic middle esophagus. (A, B) Transverse (A) and coronal (B)‐reformatted CT images show circumferential wall thickening in intrathoracic middle (subcarinal) esophagus with its posterior wall thickness of 10.5 mm. (C) PET/CT demonstrates hypermetabolic lesion at the area of CT abnormality with its SUVmax, 18.5. (D) Endoscopy depicts a 3‐cm‐sized semi‐circumferential protruding tumor with surface nodularity in the intrathoracic middle esophagus. (E) Tumor is infiltrating into the proper muscle layer (arrow) on EUS. On pathologic examination, G3 (left gastric) node was positive for cancer cells; either EUS or PET/CT failed to detect the node

The 435 patients consisted of 403 men and 32 women whose ages ranged from 31 to 90 years (mean, 64 years). Thirty‐five (8.0%) tumors were located in the upper thoracic, 169 (38.9%) were located in the middle thoracic, and 189 (43.4%) were located in the lower thoracic esophagus; the remaining tumors were located in extrathoracic cervical (n* *=* *1, 0.2%), in upper and middle thoracic (n* *=* *9, 2.1%), in middle and lower thoracic (n* *=* *28, 6.4%), and in upper, middle, and lower thoracic esophagus (n* *=* *2, 0.5%). In two patients (T1a and T1b cancers, respectively), skipped lesion were present in upper and lower thoracic esophagus. These two patients were regarded to have single T1a and T1b cancers respectively. The primary tumors were ≤T1a (Tis, 5; T1a, 126) (Figure 2) in 131, T1b in 234, and T2 (Figure 3) in 70 patients on histopathologic examination (Table 1).

Primary tumors present pathologically with N0 disease in 298 (68.5%) of 435 patients; 121 (92.4%) of 131 with ≤T1a cancers, 146 (62.4%) of 234 with T1b cancers and 31 (44.3%) of 70 with T2 eSCC. The tumors had N1 disease (Figure 3) in 99 (22.8%) patients; nine (6.9%) of 131 with ≤T1a cancers, 69 (29.5%) of 234 with T1b cancers and 21 (30.0%) of 70 with T2 eSSC. The tumors had N2 disease in 32 (7.4%) patients; none in with ≤T1a cancers, 18 (7.7%) of 234 with T1b cancers and 14 (20%) of 70 with T2 cancers. The tumors were seen with N3 disease in six (1.4%); one (0.7%) of 131 with ≤T1a cancer, one (0.4%) of 234 patients with T1b cancers and four (5.7%) of 70 with T2 eSCC (Table 2).

**Table 2:** EUS, PET/CT and pathologic N staging characteristics

Characteristics	≤pT1a	pT1b	pT2	Total	*P* value
Pathologic N staging					
N0	121	146	31	298	<.001^a^
N1	9	69	21	99	
N2	0	18	14	32	
N3	1	1	4	6	
EUS N staging					
N0	120	184	43	347	<.001^a^
N1	9	45	24	78	
N2	2	5	3	10	
N3	0	0	0	0	
PET/CT N staging					
N0	122	182	45	349	<.001^a^
N1	8	49	24	81	
N2	1	3	1	5	
N3	0	0	0	0	
Total	131	234	70	435	

a Calculated with Pearson's chi square test.

### 3.2 Diagnostic performance of preoperative T and N staging

In discriminating ≤T1a from other eSCCs, the sensitivity, specificity, and accuracy of EUS were 60.3% (79/131), 80.3% (244/304), and 74.3% (323/435), respectively, whereas those of CT were 82.4% (108/131), 37.8% (115/304), and 51.3% (223/435) respectively. In discriminating N0 from node positive disease, sensitivity, specificity, accuracy of EUS were 89.6% (267/298), 41.6% (57/137), 74.5% (324/435), respectively, whereas those of PET/CT were 88.9% (265/298), 38.7% (53/137), 73.1% (318/435) respectively.

Sensitivity, specificity, accuracy, PPV, and NPV of EUS and PET/CT for staging other T and N diseases were described in Table 3. In T staging, the differentiation of T1b cancer from other T‐stage cancers was difficult. In this occasion, using SUVmax was better than using other devices. In N staging, sensitivity was low in detecting nodal metastasis with both imaging devices.

**Table 3:** Diagnostic performance of EUS and CT or PET/CT in T and N staging

	Sensitivity	Specificity	Accuracy	PPV	NPV
T staging using EUS					
T1a	60.3% (79/131)	80.3% (244/304)	74.3% (323/435)	56.8% (79/139)	82.4% (244/296)
T1b	44.9% (105/234)	72.1% (145/201)	57.4% (250/435)	65.2% (105/161)	52.9% (145/274)
T2	47.1% (33/70)	84.4% (308/365)	78.4% (341/435)	36.7% (33/90)	89.3% (308/345)
T staging using CT					
T1a	82.4% (108/131)	37.8% (115/304)	51.3% (223/435)	36.4% (108/297)	83.3% (115/138)
T1b	21.8% (51/234)	84.6% (170/201)	50.8% (221/435)	62.2% (51/82)	48.2% (170/353)
T2	52.9% (37/70)	94.5% (345/365)	87.8% (382/435)	64.9% (37/57)	91.3% (345/378)
T staging using SUVmax^a^					
T1a	74.8% (98/131)	70.1% (213/304)	71.5% (311/435)	51.9% (98/189)	86.6% (213/246)
T1b	46.2% (108/234)	80.6% (162/201)	62.1% (270/435)	73.5% (108/147)	56.3% (162/288)
T2	77.1% (54/70)	87.7% (320/365)	86.0% (374/435)	54.5% (54/99)	95.2% (320/336)
N staging using EUS					
N0	89.6% (267/298)	41.6% (57/137)	74.5% (324/435)	76.9% (267/347)	64.8% (57/88)
N1	31.3% (31/99)	86.0% (289/336)	73.6% (320/435)	39.7% (31/78)	81.0% (289/357)
N2	9.4% (3/32)	98.3% (396/403)	91.7% (399/435)	30.0% (3/10)	93.2% (396/425)
N3	0% (0/6)	100% (429/429)	98.6% (429/435)	Indeterminate^b^	98.6% (429/435)
N staging using PET‐CT					
N0	88.9% (265/298)	38.7% (53/137)	73.1% (318/435)	75.9% (265/347)	61.6% (53/86)
N1	30.3% (30/99)	84.8% (285/336)	72.4% (315/435)	37.0% (30/81)	80.5% (285/354)
N2	0% (0/32)	98.8% (398/403)	91.5% (398/435)	0% (0/5)	92.6% (398/430)
N3	0% (0/6)	100% (429/429)	98.6% (429/435)	Indeterminate^b^	98.6% (429/435)

a T staging was performed using cut‐off value from ROC analysis (3.05 and 5.65).

b Some of PPV cannot be calculated, because there was no true positive probably due to small number of N3 disease in the cohort sample.

EUS and PET/CT missed nodal metastasis on a per‐patient basis in 80/137 (58.3%) and 84/137 (61.3%) respectively.

### 3.3 The agreement of pathologic T and N staging with EUS or CT staging

The agreement of EUS and pathologic T staging was moderate, whereas that of CT with pathologic T staging was fair (0.411 vs 0.361), but there was no significant statistical difference in T staging capability between the two modalities (Table 4).

**Table 4:** Intermodality difference between EUS and CT or PET/CT for predicting pathogic T and N staging

**EUS vs pathologic T Staging** a ^,^ c	**CT vs pathologic T staging** a ^,^ c	***P*** **value** b
0.411 (0.323‐0.484)	0.361 (0.282‐0.435)	1.000
**EUS vs pathologic N staging** a ^,^ c	**PET/CT vs pathologic N staging** a ^,^ c	***P*** **value** b
0.379 (0.269‐0.494)	0.321 (0.220‐0.418)	.660

a Values are weighted kappa with 95% confidence interval using bootstrap method.

b Calculated by generalized estimating equations (GEE).

c Strength of agreement: <0.20 (poor), 0.21‐0.40 (fair), 0.41‐0.60 (moderate), 0.61‐0.80 (good), and >0.81 (very good).

The agreement of EUS and pathologic N staging was fair and that of PET/CT and pathologic N staging was also fair (0.379 vs 0.321). There was no significant intermodality difference in N staging capability between the two modalities when applying weighted kappa coefficients at the 5% significance level (Table 4).

### 3.4 SUVmax of primary esophageal cancer and ROC curve analysis

The mean SUVmax of all primary cancers was 4.48 (range of Q1 to Q3, 2.40‐5.40); 2.53 (range of Q1 to Q3, 1.00‐3.10) in ≤T1a cancers (Figure 2), 4.02 (range of Q1 to Q3, 2.50‐4.80) in T1b cancers, 9.69 (range of Q1 to Q3, 5.78‐13.4) in T2 cancers (Table 1; Figure 3). The SUVmax was significantly different among three groups and showed correlation with pathologic T stages (*r *=* *.555, with Pearson's correlation; *P *<* *.001).

With ROC curve analysis, cut off value of SUVmax 3.05 (AUC: 0.757; 95% CI, 0.710‐0.803; *P *<* *.001) at PET provided sensitivity 74.8% (98/131), specificity 70.1% (213/304), accuracy 71.5% (311/435), PPV 51.9% (98/189), and NPV 86.6% (213/246), respectively, for differentiating ≤T1a eSCCs from other cancers. Cut‐off value of SUVmax 5.65 (AUC: 0.897; 95% CI, 0.857‐0.937; *P *<* *.001) provided sensitivity 77.1% (54/70), specificity 87.7% (320/365), accuracy 86.0% (374/435), PPV 54.5% (54/99), and NPV 95.2% (320/336), respectively, for differentiating T1 (≤T1b) eSCCs from T2 eSCCs.

## 4 DISCUSSION

Cuellar et al^12^ asserted that FDG PET/CT is not useful in the evaluation of adenocarcinoma of the esophagus when endoscopy and biopsy indicates clinical Tis and T1 in tumor stage. FDG PET/CT has no role in the evaluation of the primary tumor, detection of locoregional nodal, and distant metastatic disease in these patients. As regional nodal metastases are uncommon and distant metastases are rare in patients with superficial esophageal tumors and because FDG PET/CT could result in inappropriate patient management, FDG PET/CT should not be used in the evaluation of patients with clinical Tis and T1 esophageal adenocarcinomas. In their study, 14 (18%; pT1a [n* *=* *3], pT1b [n* *=* *9], pT2 [n* *=* *1], and pT3 [n* *=* *1]) of 79 patients had locoregional nodal metastases in resected specimen. In clinical nodal staging of these 14 patients, none of the lymph nodes were enlarged (>10 mm in diameter) on CT. FDG PET/CT was falsely negative in the 13 patients who had pN1 (12 patients) and pN2 (one).

As for T1a lesions, lymph node metastases have been variably reported but generally found to be <10% in recent series, even though most reports have dealt with esophageal adenocarcinomas rather than eSCC.^13^ In a cumulative‐data analysis, Merkow et al^13^ found that 5% of patients with T1a lesions (of 5390 patients, 10.6% eSCC) have involved lymph node metastases and 16.6% for T1b tumors. Differently Duan et al^14^ reported that lymph node metastasis rates are 17.5% for pT1 eSCC tumors, 16.0% (8 of 50) for pT1a tumors, and 22.6% (21/93) for T1b tumors. Of 25 patients with lymph node metastasis, one had cervical metastasis, 15 had thoracic metastasis, and 17 had abdominal metastasis. In our study, 10 (7.6%) of 131 with ≤T1a cancers, and 88 (37.6%) of 234 with T1b cancers had nodal metastases. The rates were similar to those in a previous report published at our institution, where the rates of lymph node involvement were 6.25% (4 of 64) in T1a cancers and those of T1b cancers were 29.3% (39 of 133) (*P *<* *.001).^15^ Thus, In ≤T1a eSCCs, lymph node metastases were much lesser than those in the study of Duan et al,^14^ whereas those were much higher in T1b esophageal cancer. Given the context that the management could be ESD in T1a esophageal cancers, the lymph node metastasis rates in our study appear to be more applicable and reliable.

Comparison of FDG uptake in the primary tumor and clinicopathologic staging findings showed that there is a significant association between the FDG uptake in the primary tumor and depth of invasion.^16^ In our study, when applying SUVmax 3.05 as cutoff point in discriminating ≤T1a from higher stage cancers, sensitivity, specificity, and accuracy were 74.8% (98/131), 70.1% (213/304), and 71.5% (311/435) respectively. The results were comparable with the performance of EUS where the sensitivity, specificity, and accuracy were 60.3% (79/131), 80.3% (244/304), and 74.3% (323/435) respectively.

Endoscopic ultrasonography has some limitations in T staging, because EUS is not an option for evaluating stenotic tumors. Stenotic esophageal cancers account for 30% of the cancers. However, in our case series of T1a‐T2 stages, stenotic tumors hampering the passage of endoscopic probe were absent. EUS also has some difficulty in nodal staging when the target nodes are not accessible.^17^

In our study, T staging in both EUS and CT showed only less than moderate degree of agreement with pathologic T staging and there was no intermodality difference between EUS and CT or PET/CT for predicting the pathologic T staging. PET/CT showed high sensitivity in particular for differentiating ≤T1a eSCCs from higher‐stage cancers by noticing no tumor on CT and by measuring SUVmax of ≤3.05 in patients with an esophageal cancer seen on endoscopy. We think the criteria of no identifiable tumefaction on CT and little FDG uptake of <3.05 at PET help discriminate the ≤T1a cancers, thus, allowing us to select candidates for ESD.

N staging in both EUS and PET/CT demonstrated only fair agreement with pathologic N staging, and there was no significant difference in their N staging performance between the two modalities. Therefore, it would provide interesting prognostication results to compare survival between those showing N0 disease in clinical staging but positive nodal metastasis in pathologic examination and those demonstrating true clinical and pathologic N0 stage.

In our study, the sensitivity, specificity, accuracy, PPV, and NPV of PET/CT in discriminating N0 from node positive disease in eSCC patients of both T1 and T2 stage were 88.9% (265/298), 38.7% (53/137), 73.1% (318/435), 75.9% (265/347), and 61.6% (53/86), respectively, whereas those of EUS were 89.6% (267/298), 41.6% (57/137), 74.5% (324/435), 76.9% (267/347), and 64.8% (57/88) respectively. However, still both PET/CT and EUS miss nodal metastasis detection in more than half of the patients; 84/137 (61.3%) in PET/CT and 80/137 (58.3%) in EUS respectively. It was stated that median lymph node size in missed lymph node metastasis was 3 mm in diameter, with 41 (82%) of 50 metastatic lymph nodes of <6 mm.^18^

Our study suffered from several limitations. First, our study design was retrospective thus we might have selection bias; (1) our study population was composed of surgically confirmed esophageal cancer patients only and (2) although we tried to include as many as patients with a surgically resectable eSCC, 297 (40.6%) of 732 patients having surgical resection were excluded owing to various reasons. Second, it was conducted only in a single tertiary referral hospital. Third, there were only few N3‐stage patients in our patient cohort where only pathologic T1‐ and T2‐stage eSCC were included. Therefore, diagnostic performance of EUS and PET/CT for high nodal‐stage disease, particularly the PPV for N3 disease, may have been inaccurate.

In conclusion, with SUVmax cut‐off value of 3.05, differentiation of T1a from higher T stage cancers can be achieved in >70% of surgically resectable eSCCs and PET/CT and EUS provide comparable performance in differentiating N0 eSCCs from node metastasis‐positive cancers. Substantial percentage (7.6%) of ≤T1a eSCC patients have nodal metastases, and nodal metastasis rates increase as T stage increases (T1b [37.6%] and T2 [55.7%]). Moreover, more than half of nodal metastases were missed on PET/CT or EUS. Thus, after endoscopic surgery or even after curative surgical resection of <T1a eSCCs, adjuvant therapy is needed for those having nodal metastasis.

## CONFLICT OF INTEREST

None declared.
