# Supplementary material for: Surgically resected T1‐ and T2‐stage esophageal squamous cell carcinoma: T and N staging performance of EUS and PET/CT
Source: Cancer Med. 2018 Jun 22;7(8):3561–70. doi: 10.1002/cam4.1617 (PMC6089181; doi:10.1002/cam4.1617)
Supplement: Supplementary file 1 [file CAM4-7-3561-s001.docx]

**Supplemental Data**

**Materials and Methods**

**CT and PET/CT Scanning**

CT scanners for preoperative enhanced chest CT were Light Speed VCT, Light Speed Ultra, Light Speed QX/i, and Discovery CT750 HD (GE Healthcare, Chalfont St Giles, England); Somatom Definition Flash, (Siemens Medical Solutions, Forchheim, Germany); Brilliance 40 (Philips, Best, the Netherlands); Aquilion 64 (Medical Systems, Otawara, Japan).
